# Acute Bacterial Prostatitis Caused by Staphylococcus saprophyticus: A Case Report

**DOI:** 10.7759/cureus.64187

**Published:** 2024-07-09

**Authors:** Christopher T Gabbert, Fariha Bhuiyan, Intekhab Askari Syed

**Affiliations:** 1 Medical School, Sentara Halifax Regional Hospital Clinical Site, Edward Via College of Osteopathic Medicine, Blacksburg, USA; 2 Family Medicine, Sentara Halifax Regional Hospital Clinical Site, Edward Via College of Osteopathic Medicine, Blacksburg, USA; 3 Family Medicine, Sentara Clarksville Family Medicine, Clarksville, USA

**Keywords:** lower urinary tract symptoms, urinary track infection, acute bacterial prostatitis, staphylococcus saprophyticus, male urology

## Abstract

Acute bacterial prostatitis can burden patients with an abrupt onset of lower urinary tract symptoms. Proper treatment is necessary to prevent various complications that require hospitalization and surgical intervention. Thus, it is important to know what bacteria may cause this infection and what treatments may lead to a complete resolution. While acute bacterial prostatitis is usually caused by* Escherichia coli, Enterobacteriae* species, and various other species, *Staphylococcus saprophyticus* is a relatively unique cause that has seldomly been associated with any prostatic diseases. This case involves a 46-year-old Caucasian male with no previous history of prostate diseases who presented to the clinic with fevers, chills, diarrhea, and resolved urinary symptoms. Upon further clinical workup, the patient was found to have an elevated prostate-specific antigen level, along with a positive urinary culture for *Staphylococcus saprophyticus*. Following seven days of antibiotic treatment, prostate-specific antigen levels had significantly decreased, and the patient’s symptoms had fully resolved. No further symptoms were noted after the completion of the full 28-day course of antibiotics. This paper explores how the patient’s social, medical, and surgical history may have led to this type of infection. Focus will be placed on areas of research that need to be extended for future cases of acute bacterial prostatitis caused by *Staphylococcus saprophyticus*. This case intends to inform future clinical practice by identifying predisposing factors to prevent occurrence and by discussing treatment strategies to achieve infection resolution.

## Introduction

Prostatitis is considered a relatively common disease, with estimates suggesting a lifetime risk for men above 25% [1]. It can be divided into several categories, including acute bacterial, chronic bacterial, chronic, and asymptomatic inflammatory prostatitis [1,2]. This article will specifically discuss acute bacterial prostatitis (ABP), which accounts for approximately 1%-10% of all prostatitis cases [1,3]. Although ABP is considered less prevalent than other subtypes of prostatitis, it is important to properly recognize and diagnose this disease to prevent various complications, such as systemic infection, severe urinary retention, abscess formation, and chronic pelvic pain [3-5].

ABP is mainly thought to arise from ascending urinary tract infections (UTI), although direct inoculation is possible through transurethral and prostatic surgical interventions [1,3]. Appropriately, risk factors include behaviors that increase UTI prevalence, along with anatomical/surgical anomalies and immunocompromised states [3]. Complications are more probable in cases where antibiotic treatments have been unsuccessful [5,6]. Furthermore, the rate at which ABP can become chronic is unclear, although one cohort study of 437 men found that resolution of disease was attained by 82% of subjects without extension to a chronic form [4,7].

ABP is primarily caused by* Escherichia coli* infections, followed by *Enterobacteriae* species, *Enterococcus* species, *Pseudomonas aeruginosa*, and rarely bacteria that usually cause sexually transmitted infections (STI) [1,3]. Clinical presentation includes a variety of urinary symptoms, such as dysuria, urgency, increased frequency, hesitancy, and incomplete voiding, along with sexual symptoms including ejaculatory discomfort [1,3]. Evaluation must take sexual history and immune status into consideration because these are both factors that can predispose patients to atypical bacteria [3]. Additionally, diagnosis is achieved through proper physical and clinical findings. Physical exam findings may include acute fever and pelvic tenderness to palpation [1]. Prostate massage and the 4-glass test have been largely replaced with laboratory work, as it is thought that direct manipulation can increase the risk for urosepsis in ABP cases [3,4]. Laboratory findings may include elevated prostate-specific antigen (PSA) levels, positive urinalysis (UA) and urinary cultures, and possibly positive blood cultures in severe cases [3].

Mild cases of ABP can be treated outpatiently with several oral antibiotic regimens that include fluoroquinolones and trimethoprim-sulfamethoxazole (TMP-SMX) [1,3]. In cases where sepsis, abscess formation, and other complications are considered likely, stronger parenteral regimens are often required [1]. Furthermore, all treatments are also guided by culture sensitivities and local resistance rates. While regimens are typically limited to a two-week duration to prevent resistance and antibiotic-related side effects, up to four weeks may be required for infection clearance [1,3,6]. Overall, treatment is guided by the incorporation of specific prognostic factors derived from history, physical findings, and laboratory results.

This case involves a patient who acquired ABP from *Staphylococcus saprophyticus*. This etiology is quite unusual, as *S. saprophyticus* is usually associated with lower UTIs in sexually active young women [8]. Given the rarity of this occurrence, it is important to explore potential reasons for this infection, as it can inform future preventative and treatment strategies.

## Case presentation

In November, a 46-year-old Caucasian male with a history of obesity, hypertension, hypothyroidism, low testosterone, and sleep apnea presented with his wife to a rural clinic complaining of fever for the past five days. He attributed his fever to a recent influenza vaccination received in the week prior, which delayed him from seeking medical attention. His highest recorded temperature at home was 102 ºF (38.9 ºC). He had been managing his symptoms with acetaminophen 650 mg and ibuprofen 200 mg, as needed. The patient also reported symptoms of chills, headaches, and diarrhea at this time. He denied shortness of breath, cough, nausea, vomiting, myalgias, and arthralgias. During this five-day febrile period, the patient also had urinary urgency with an inability to void; no dysuria, urinary frequency, or trouble ejaculating were reported. These urinary symptoms had already been resolved by the time he was seen in the clinic. No other known history of UTI or prostate disease was noted at the time. The patient was in a monogamous relationship with his wife. The wife also denied recent sickness, urinary tract/fungal infection symptoms, and diarrhea.

Regarding his general medical history, the patient started semaglutide for weight loss two months prior to the encounter and was currently using 2.4 mg/0.75 mL subcutaneous injections weekly. He was managing his blood pressure with a low-sodium diet. He recently started taking daily levothyroxine 50 µg two weeks prior. Furthermore, following a testosterone level measurement below 200 ng/mL, he started daily usage of 1.62% topical testosterone for the past two months. Additionally, he was compliant with his continuous positive airway pressure device for several years.

His vital signs and physical exam were generally noncontributory. The patient’s temperature was 98.7 ºF (37.1 ºC) with a heart rate of 98 bpm and a blood pressure of 110/60 mmHg. Pulse oximetry read 96% on room air. His body mass index (BMI) was 47.42 kb/m^2^ at the time. Among other findings, the patient was in no acute distress; his mucous membranes were moist; no adventitial lung sounds were noted; and his abdomen was non-tender. At this time, the patient was nasally swabbed for flu and COVID; both rapid tests came back negative. The patient provided a midstream clean-catch urine sample for analysis and culture, along with a blood sample for PSA screening (Table 1). The patient was sent home with a prescription for TMP-SMX 160-800 mg to be taken twice daily for 10 days.

**Table 1:** Results of urinalysis, culture, and prostate-specific antigen screening.

Parameter	Initial Visit	One-Week Follow-Up
Color	Yellow	Light yellow
Clarity (clear, slightly cloudy)	Clear	Clear
Specific gravity (1.005–1.030)	1.015	1.021
pH (5.0–8.0)	6.5	5.5
Protein screen (negative/trace, mg/dL)	100	Negative
Glucose (negative, mg/dL)	Negative	Negative
Ketones (negative, mg/dL)	Negative	Negative
Bilirubin (negative)	Negative	Negative
Urobilinogen ( 0.2–2.0 mg/dL)	0.2	0.2–2.0
Blood (negative)	Small	Trace
Nitrite (negative)	Negative	Negative
Leukocyte esterase (negative)	Small	Negative
Urine culture (<10,000 organisms/mL)	>100,000 (*S. saprophyticus*)	No growth
Prostate-specific antigen (<2.0 mg/dL)	32.40	10.10

Urinalysis was remarkable for blood and leukocyte esterase; his culture returned positive for *S. saprophyticus*, and PSA returned as 32.4 ng/mL (Table 1). Two months prior, the patient had a PSA of 0.823 ng/mL. It was at this time that the patient was contacted with a working diagnosis of ABP. He was advised to continue using TMP-SMX 160-800 mg for 28 days from the initial encounter. The patient was also told to return in seven days for additional laboratory work and symptom monitoring.

Upon returning for the one-week follow-up, the patient presented with no further symptoms. The fever had resolved, UA showed trace blood, PSA was down to 10.1 ng/mL, and urine cultures returned with no growth after one day (Table 1). The patient was counseled regarding the need to adhere to his prescribed antibiotic treatment and was advised to seek medical attention if any more symptoms developed. The patient reported no future recurrence. He returned to the clinic weeks later with no further complaints.

## Discussion

*S. saprophyticus* is a bacterium that can establish itself in the urothelial tract through a variety of mechanisms. Specifically, it possesses adhesion proteins that bind well to urothelial cells, can form a biofilm, and contains urease to help it maintain favorable pH conditions for growth [8]. With *S. saprophyticus* urogenital infections, the bacteria are typically found within the rectal flora before extending into the genitourinary flora [8-10]. While it is considered the second most common cause of uncomplicated cystitis in young, sexually active women, it has been known to cause several types of genitourinary infections in men [9]. However, the prevalence of these male-specific infections is quite unclear. Upon a broad literature search, there have only been a few reported cases of *S. saprophyticus* causing acute or chronic bacterial prostatitis [11-13]. These articles vary in terms of patient demographics, with predispositions for infections seemingly discordant and dependent upon previous antibiotic use and chronicity [11-13]. With that said, it is important to thoroughly characterize cases where *S. saprophyticus* does cause ABP in order to prevent future incidences.

In this case, the patient reported being in a monogamous relationship with his wife. *S. saprophyticus* could reasonably be spread from a sexual partner, as it has been estimated to reside in the normal flora of genitourinary tracts in up to 40% of asymptomatic women [8]. Furthermore, women are more likely to develop *S. saprophyticus* infections following recent sexual intercourse and candidiasis infections [9]. The patient’s wife denied recent UTI and fungal infections, which makes spreading through sexual contact less likely. Yet, given that *S. saprophyticus* can reside normally in sexually active women, it cannot be ruled out completely.

The patient’s medical history could also give insight into how he may have contracted this infection. He had been recently diagnosed with low testosterone, with his total testosterone measuring below 200 ng/dL two months prior to the infection. There is literature that suggests testosterone levels are inversely correlated with symptom severity in cases of chronic prostatitis [14]. Furthermore, testosterone supplementation has been shown to be beneficial to hypogonadal patients with chronic forms of prostatitis [15]. While these pieces of literature refer to chronic forms of prostatitis, they may give insight into the relationship between prostatitis development and total testosterone levels. Concerning acute forms of prostatitis, testosterone supplementation has been shown to reduce *E. coli* invasion and subsequent prostatitis severity [16]. Overall, more research is necessary to determine if low testosterone is truly a risk factor for ABP and whether supplementation may influence severity and complication rates.

Regarding the patient’s weight, obesity is known to be a risk factor for several prostate diseases, including benign prostate hyperplasia and prostate cancer [17]. However, it has not been proven to be a risk factor for prostatitis [17]. Interestingly, there may be some association between thyroid disease and the risk of prostatitis. Elevated thyroid-stimulating hormone (TSH) levels have been associated with a reduced risk of genetically predicted prostatitis [18]. This relationship can be explained theoretically, as primary hypothyroidism may lead to hypogonadotropic hypogonadism through hypersecretion of prolactin. Therefore, if there was truly an association between low testosterone and prostatitis, there could also be an indirect association with hypothyroidism as well.

Outside of medical and social history, the patient did not have pertinent surgical history, such as transurethral procedures, recent urethral catheterization, or prostatic biopsies.

The workup for this patient resulted in markedly elevated PSA levels and a positive UA, with urinary cultures, demonstrating growth of *S. saprophyticus*. Given his history of acute symptom onset, chronic prostatitis was considered a less likely diagnosis. Furthermore, the patient’s sexual history and urinary-specific symptoms made orchitis and epididymitis less likely even prior to laboratory confirmation. Additionally, lack of suprapubic tenderness made cystitis less likely.

Once the patient was formally diagnosed with ABP, he began showing marked improvement with the cessation of symptoms after one week of antibiotics and a significant reduction in follow-up PSA levels from 32.4 mg/dL to 10.1 mg/dL. The choice to start the patient on TMP-SMX served three-fold, as it covered urethritis, cystitis, and prostatitis prior to laboratory confirmation. TMP-SMX is considered an appropriate antibiotic for ABP in areas where resistance is below 10%-20%, as trimethoprim can reach high levels of concentration in the prostate [1,2,6]. No alpha-1 antagonists were considered since the patient experienced no urinary obstructive symptoms at the time of diagnosis.

Overall, the patient recovered well, and no further recurrences had been reported in the months following his treatment, suggesting that full resolution was achieved.

## Conclusions

The patient in this case was found to have an uncomplicated course of ABP caused by *S. saprophyticus*, where resolution was achieved through a 28-day course of TMP-SMX. It is uncertain how the patient may have acquired this infection. However, his low testosterone state and low thyroid state, along with his sexual history with a female partner, may have played a role. This case highlights the need for further examination of ABP caused by *S. saprophyticus*. The current research points toward investigation into *S. saprophyticus* virulence factors, the overall prevalence of *S. saprophyticus* in non-pathological conditions, and patient factors such as testosterone and thyroid levels. In summation, the findings of this case can inform future practice regarding similar prostatitis infections and can help elucidate attenuable predisposing factors.
